# Suppressive Effect on Lipopolysaccharide-Induced Proinflammatory Mediators by *Citrus aurantium L.* in Macrophage RAW 264.7 Cells via NF-*κ*B Signal Pathway

**DOI:** 10.1155/2011/248592

**Published:** 2010-09-21

**Authors:** Sang-Rim Kang, Dae-Yong Han, Kwang-Il Park, Hyeon-Soo Park, Yong-Bae Cho, Hu-Jang Lee, Won-Sup Lee, Chung Ho Ryu, Yeong Lae Ha, Do Hoon Lee, Jin A. Kim, Gon-Sup Kim

**Affiliations:** ^1^Institute of Life Science and College of Veterinary Medicine, Gyeongsang National University, Gazwa, Jinju 660-701, Republic of Korea; ^2^Department of Internal Medicine, Institute of Health Sciences, Gyeongsang National University School of Medicine and Gyeongnam Regional Cancer Center, Gyeongsang National University Hospital, Jinju 660-702, Republic of Korea; ^3^Food Technology, Institute of Agriculture & Life Science, Gyeongsang National University, Jinju 660-701, Republic of Korea; ^4^Division of Applied Life Sciences (BK21 program), Graduate School and Institute of Agriculture & Life Science, Gyeongsang National University, Jinju 660-701, Republic of Korea; ^5^Korea National Animal Research Resource Center and Korea National Animal BioResource Bank, Gyeongsang National University, Jinju 660-701, Republic of Korea

## Abstract

Citrus fruits have been used as an edible fruit and a traditional medicine since ancient times. In particular, the peels of immature citrus fruits are used widely in traditional herbal medicine in Korea, as they are believed to contain bioactive components exerting anti-inflammatory activity. This study examined whether the crude methanol extract of *Citrus aurantium L.* (CME) has a suppressive effect on inducible enzymes and proinflammatory cytokines by inhibiting the NF-*κ*B pathway in LPS-stimulated macrophage RAW 264.7 cells. The cells were pretreated with the indicated concentrations of CME (5, 10, 20, and 50 *μ*g/mL) and then treated with LPS (1 *μ*g/mL). The results showed that CME (10, 20, and 50 *μ*g/mL) inhibited the LPS- (1 *μ*g/mL) induced mRNA and protein expression of iNOS in macrophage Raw 264.7 cells. In addition, the expression of COX-2 was inhibited at the mRNA and protein levels by CME in a dose-dependent manner. The mRNA expression of proinflammatory cytokines, such as TNF-*α* and IL-6, were markedly reduced by CME (10, 20, and 50 *μ*g/mL). Moreover, CME clearly suppressed the nuclear translocation of the NF-*κ*B p65 subunits, which was correlated with its inhibitory effect on I-*κ*B phosphorylation. These results suggest that CME has anti-inflammatory properties by modulating the expression of COX-2, iNOS, and proinflammatory cytokines, such as TNF-*α* and IL-6, in macrophage RAW 264.7 cells via the NF-*κ*B pathway.

## 1. Introduction

*Citrus aurantium L. *(bitter orange) is a flowering plant that belongs to the Rutaceae family of the order* Sapindales*, and is distributed widely in tropical and subtropical southeast regions of the world. The dried, entire immature peels of citrus fruit are used as a traditional herbal medicine to treat indigestion and respiratory symptoms, such as bronchial and asthmatic conditions [1, 2]. Citrus fruit including *C. aurantium L.* is composed of several components, such as limonoid, polyphenols, and flavonoids [3]. The major flavonoids isolated from *Citrus aurantium L.* are hesperidin, naringenin, and nobiletin [4]. These flavonoids have the ability to regulate the inflammatory response and carcinogenesis at many key regulatory points through a variety of mechanisms [5]. Nobiletin isolated from Citrus spp. has an inhibitory effect on phorbol ester-induced skin inflammation [6, 7]. In addition, it was reported that hesperidin can inhibit the expression of lipopolysaccharine- (LPS-) induced cyclooxygenase (COX-2) and inducible nitric oxide (iNOS) in macrophage RAW 264.7 cells [8]. Therefore, this study examined the anti-inflammatory effect of the dried peel of *Citrus aurantium L.*

Inflammation initiated by the invasion of pathogens or cell injury is a normal physiological and immune response. The normal inflammatory response involves the activation of several different cellular components, such as macrophages, neutrophils, and lymphocytes. In particular, macrophages play an important role in modulating inflammation and the immune response to maintain a defensive reaction [9, 10]. During the inflammatory response, activated macrophages secrete large amounts of proinflammatory mediators, such as nitric oxide (NO) and prostaglandinE2 (PGE2), via the inducible isoforms of NO synthase (iNOS) and cyclooxygenase-2 (COX-2), respectively, as well as proinflammatory cytokines, such as TNF-*α*, IL-6, and IL-1*β* [11, 12]. 

Most NO is synthesized through the oxidative deamination of L-arginine by a family of nitric oxide synthase (NOS) in mammalian cells [13]. These NOSs are found in three isoforms, neuronal NOS (nNOS), endothelial NOS (eNOS), and inducible NOS (iNOS). In particular, during the inflammatory response, iNOS is expressed in response to endotoxin, such as LPS and various proinflammatory cytokines including INF-*γ* and TNF-*α* [14]. In addition, NO produced by iNOS is regarded as a critical mediator of carcinogenesis and high levels of NO cause inflammatory diseases, such as atherosclerosis, bowel disease, rheumatoid arthritis, and septic shock [15–18].

Similar to iNOS, cyclooxygenase (COX) is a key enzyme catalyzing the formation of prostaglandin from arachidonic acid during the inflammatory reaction [19]. There are two types of cyclooxygenase, COX-1 and COX-2. COX-1 is expressed constitutively in most cells and believed to be involved in homeostatic prostanoid biosynthesis. In contrast, COX-2 is not expressed in most normal tissues but is increased rapidly by oncogenes, growth factors, and cytokines [20]. COX-2 is involved in many biological processes, including inflammation, tumorigenesis, and carcinogenesis [21]. Briefly, the overexpression of proinflammatory mediators, such as iNOS, COX-2, and various cytokines, is involved in the pathogenesis of many disease processes. Therefore, the targeted inhibition of iNOS and COX-2 is a promising approach to inhibiting inflammation and carcinogenesis, as well as preventing cancer. 

**Figure 1:**
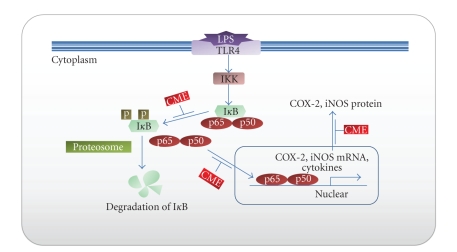
Main molecular targets of CME that lead to inhibitory effects on LPS-induced inflammation action. CME blocking NF-*κ*B signaling pathway via inhibition of (i) I*κ*B phosphorylation, (ii) subunits (p65/p50) of NF-*κ*B trnaslocation in nuclear, and (iii) proinflammatory mediators (COX-2, iNOS, etc.) transcription. Blue arrows indicate the NF-*κ*B signal pathway and target gene that DNA binding site of NF-*κ*B. Red boxes indicate the inhibition effect of CME. CME: Crude methanol extract of *Citrus aurantium L.*; IKK: I*κ*B kinase; I*κ*B: Inhibitor of *κ*B in cytoplasm; p50/p65: subunits of NF-*κ*B; COX-2: cyclooxygenase-2; iNOS: inducible nitric oxide synthase.

 Nuclear transcription factor kappa-B (NF-*κ*B) is a critical key transcription factor that expresses the genes involved in inflammation. NF-*κ*B is composed of a range of homo or heterodimeric combinations of NF-*κ*B/Rel proteins, such as Rel (cRel), RelA (p65), RelB, NF-*κ*B1 (p50), and NF-*κ*B2 (p52) in mammals. The main inducible form is a heterodimeric consisting of the p50/p65 subunit. NF-*κ*B is present in the cytoplasm as an inactive complex associated with an inhibitory protein called I*κ*B. Various external or internal stimuli cause the dissociation of the NF-*κ*B/I*κ*B complex through the phosphorylation and degradation of I*κ*B by cytoplasmic I*κ*B kinase (IKK). After activation, the NF-*κ*B heterodimer (p65/p50) is translocated rapidly from the cytoplasm to the nucleus (Figure 1). In the nucleus, p65/p50 of the NF-*κ*B subunit binds to a specific DNA motif and modulates the transcription of target genes including proinflammatory mediators, such as iNOS, COX-2, various cytokines, chemokines, and adhesion molecules [22–24].

 Previous studies suggested that the activation of NF-*κ*B triggers the transcription of iNOS, COX-2, and various proinflammatory cytokines, such as IL-6, IL-1, and TNF-*α* [25]. 

Therefore, the objective of this study is to elucidate the anti-inflammatory effects of crude methanol extract of *Citrus aurantium L.* on the expression of iNOS, COX-2, IL-6, and TNF-*α* via blocking of NF-*κ*B signal pathway in LPS-stimulated macrophage RAW 264.7 cells. Thus, we investigated the following: (1) the inhibitory effect of CME on the LPS-induced iNOS and COX-2 at the mRNA and protein levels; and (2) the inhibitory effect of CME on the LPS-induced proinflammatory cytokines, such as TNF-*α* and IL-6.

## 2. Methods

### 2.1. Chemicals

The following reagents were acquired commercially: RPMI 1640 from HyClone (Logan, Utah, USA); Fetal bovine serum (FBS) and antibiotics (streptomycin/penicillin) from Gibco (BRL Life technologies, Grand Island, NY,); Lipopolysaccharide (*Escherichia coli* O111:B4) and methyl thiazolyl tetrazolium (MTT) from Sigma (St Louis, MO 63103, USA).

### 2.2. Antibodies

Antibodies for iNOS, COX-2, and NF-*κ*B p65 were purchased from Santa Cruz Biotechnology (Santa Cruz, CA,). *β*-actin antibody was purchased from Chemicon International (Temecula, CA,) and phospho-I*κ*B antibody was purchased from Cell Signaling (Beverly, MA, USA).

### 2.3. Cell Culture and Treatment

The murine macrophage RAW 264.7 cell line (KCKB 40071) was purchased from the Korean Cell Line Bank (KCLB, Seoul, Korea). Mouse macrophage cell line RAW 264.7 was cultured in RPMI 1640 medium containing 10% fetal bovine serum, 100 U/ml penicillin, and 100 *μ*g/*μ*L streptomycin at 37°C in an atmosphere containing 5% CO_2_. The cells cultured in 6 well-plates were pretreated with the indicated concentrations of CME for 2 hours in the presence or absence of LPS (1 *μ*g/mL).

### 2.4. Preparation of Herbal Extracts

*Citrus aurantium L.* was purchased from ABRB (Animal Bio-Resources Bank). The extract of* C. aurantium L.* was prepared using a modified method reported by Jung et al. [3]. Briefly, the powdered peel of *C. aurantium L.* (20 g) was extracted in 200 mL of absolute methanol at room temperature for 48 hours. The methanolic extracts were filtered through filter paper, evaporated *in vacuo*, and lyophilized to give a powdered extract. The yield of the citrus methanol extract was 10% (w/v). The concentrated CME was dissolved in PBS (pH 7.4) and stored at −20°C until needed.

### 2.5. MTT Assay

The cell viability was measured using a 3-(4, 5-dimethyethiazol-2-yl)-2, 5-diphenyltetrazolium bromide (MTT) assay. The cells were seeded in a 6-well plate and incubated for 24 hours. The macrophage cells were treated with various concentrations of CME and incubated for 18 hours. 200 *μ*L of a MTT solution (5 mg/mL in a PBS) was added to the wells and incubated for three hours. After media suction, 500 *μ*L of dimethyl sulfoxide (DMSO) was added to each well to dissolve the crystalline deposits on the cells. The optical density of the cells at 540 nm was measured using an ELISA plate reader.

### 2.6. Western Blot

The cells were cultured in 6-well plates and incubated with CME (various concentrations) in the presence or absence of LPS (1 *μ*g/mL) for 18 hours. After washing with ice-cold PBS, the cells were lysed in a buffer [50 mM Tris-Hcl (pH 8.0), 150 mM NaCl, 0.5% sodium deoxycholate, 0.1% SDS, and 1% NP-40] containing the protease inhibitor cocktail, 0.5 M EDTA, and phosphatase inhibitor at a final concentration of 1X. The cell lysates were centrifuged at 13,000 rpm for 30 minutes to remove the debris. The protein concentration was determined using Bradford assay (Bio-rad, Hercules, CA, USA). The entire cell lysates were separated by 10% SDS-PAGE and transferred to a polyvinylidene fluoride (PVDF) membrane (Immunobilon-P, 0.45 mm; Millipore, USA) using the TE 77 Semi-Dry Transfer Unit (CE Healthcare Life Sciences). The membranes were blocked with 5% nonfat skim milk in Tris buffered saline containing 0.5% Tween-20 (TBS, pH 7.4) at room temperature for 30 minutes, and incubated overnight at 4°C with the antibodies to iNOS, COX-2, *β*-actin, p-I*κ*B, and NF-*κ*B p65. The membranes were washed four times with TBS-T for 10 minutes each and incubated with a 1 : 2000 dilution of horseradish peroxidase-conjugated secondary antibody for 1 hour. The membranes were then rewashed five times with TBS-T. The proteins were visualized using an enhanced chemiluminescence kit (ECL), Western Blotting Detection Reagents (GE Healthcare Life Sciences), and exposed to X-ray film (Fuji, Tokyo, Japan). Then each band was quantitatively determined using Image J, and the density ratio represented the relative intensity of each band against those of *β*-actin as a control in each experiment.

### 2.7. Reverse-Transcription Polymerase Chain Reaction (RT-PCR)

The total RNA was isolated using a TRIzol reagent (GeneALL Biotechnology CO., LTD, KOREA), according to the manufacturer`s instructions. The total RNA (1 *μ*g) was reverse-transcribed into cDNA using commercially available cDNA synthesis kits (iScript cDNA Synthesis Kit; BIO-RAD). The tubes were incubated at 25°C for 5 minutes and then at 42°C for 30 minutes followed by heating at 85°C for 5 minutes. The sample was then stored at −70°C until further analysis. The PCR primers used with mouse macrophage RAW 264.7 cell cDNA are listed in Table 1. The PCR primers used to amplify the mouse COX-2, NOS2, TNF-*α*, IL-6, and GAPDH cDNA were purchased from Bioneer Corporation Inc. After initial heat denaturation at 95°C for 4 minutes., the PCR cycles were repeated 30~35 times under the following conditions: denaturation at 95°C for 30 seconds, annealing at 52~55°C for 1 minute, and extension at 72°C for 1 minute. The amplified PCR products were stored at −20°C until analysis. The PCR products were analyzed on ethidium bromide-stained 1.5% agarose gels. The amount of mRNA was evaluated by densitometry. The signal intensity of the specific mRNAs were normalized by a comparison with that of GAPDH and calculated as the relative amounts.

### 2.8. Statistical Analysis

The data is presented as the mean ± SD of the results of at least three experiments. One-way analysis of variance (ANOVA) was used for multiple comparison. A *P*-value <.05 was considered significant.

## 3. Results

### 3.1. Cell Cytotoxicity and Morphology of Macrophage RAW 264.7 Cells

**Figure 2:**
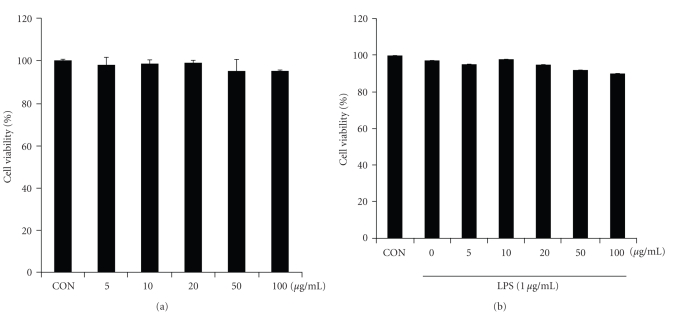
Effect of CME on the cell viability via an MTT assay. The cells were incubated with CME in the (a) absence or (b) presence of LPS for 18 hours. The results are reported as a percentage compared to the untreated controls. The data is reported as the mean ± SD of triplicate experiments.

**Figure 3:**
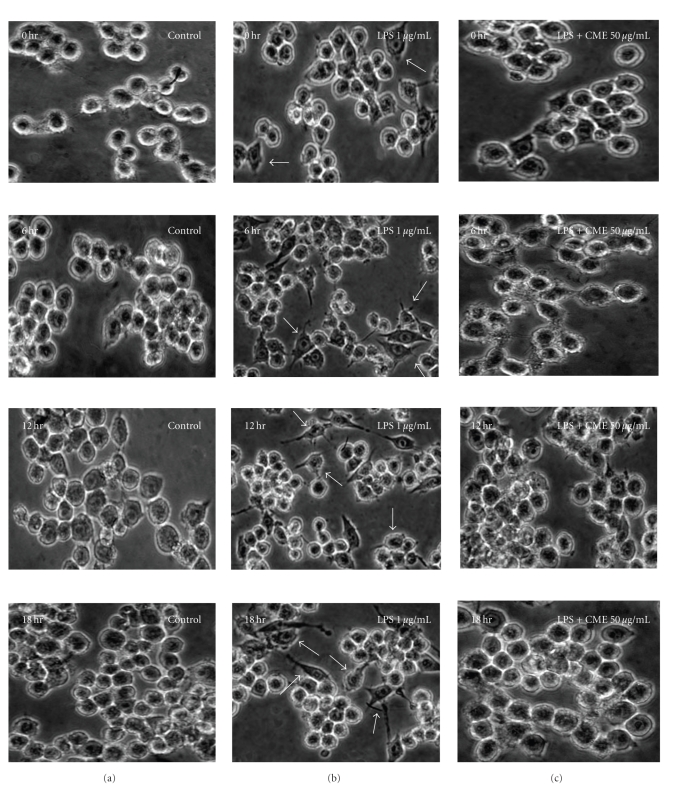
Morphological change in macrophage RAW 264.7 cells. Morphology of macrophage RAW 264.7 cell visualized by optical microscopy (×400). The cells were pretreated with CME before incubation with LPS for 18 hours. (a) Control, (b) LPS- (1 *μ*g/mL) treated only, and (c) LPS-treated with CME (50 *μ*g/mL).

The cytotoxicity of CME was examined by a MTT assay in the presence or absence of LPS (1 *μ*g/mL) to determine the effective concentration for treatment.  Figure 2 shows the cell viability at various CME concentrations (5, 10, 20, 50, and 100 *μ*g/mL) and cotreated LPS (1 *μ*g/mL) for 18 hours. The results showed that LPS (1 *μ*g/mL) and CME had no cytotoxicity at any of the concentrations examined.  Figure 3 presents the cell morphology of the macrophage RAW 264.7 cells under CME treatment in the presence or absence of LPS (1 *μ*g/mL). The cells were monitored under optical microscopy (400×) for 18 hours; see Figure 3. In the LPS-unstimulated cells, the cell morphology generally showed a round form the in whereas LPS-activated RAW 264.7 cells had changed to an irregular form with accelerated spreading and forming pseudopodia. The cotreatment of LPS with CME reduced the level of cell spreading and pseudopodia formation by suppressing cell differentiation.

### 3.2. CME Decreases LPS-Induced mRNA Expression of Proinflammatory Cytokines in RAW 264.7 Cells

**Figure 4:**
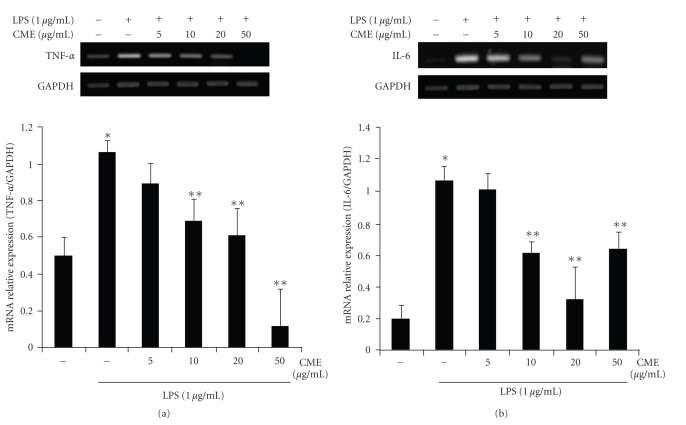
Suppressive effect of CME on the production of LPS-induced proinflammatory cytokines in RAW 264.7 cells. The RAW 264.7 cells were pretreated with CME (5, 10, 20, and 50 *μ*g/mL) for 1 hour, and then stimulated with LPS (1 *μ*g/mL). After 6 hours, the total mRNA was isolated, and the mRNA levels of (a) TNF-*α* and (b) IL-6 were examined by RT-PCR. * indicates an increase in mRNA expression relative to the control (*P* < .05), ** indicates a decrease in mRNA expression relative to the LPS group (*P* < .05).

To examine the effect of CME on LPS-induced IL-6 and TNF-*α* gene expression, the RAW 264.7 cells were pretreated with various doses (5, 10, 20, and 50 *μ*g/mL) of CME for 1 hour followed by a LPS (1 *μ*g/mL) treatment for 6 hours. The level of IL-6 and TNF-*α* gene expression in the RAW 264.7 cells was increased markedly by the LPS treatment (1 *μ*g/mL). The level of LPS-induced IL-6 and TNF-*α* gene expression decreased significantly on pretreatment with CME in the dose-dependent manner (Figure 4). This suggests that CME inhibited the mRNA expression of IL-6 and TNF-*α* in LPS-stimulated RAW 264.7 cells at the transcription levels.

### 3.3. CME Decreases LPS-Induced mRNA Expression of COX-2 and iNOS in RAW 264.7 Cells

**Figure 5:**
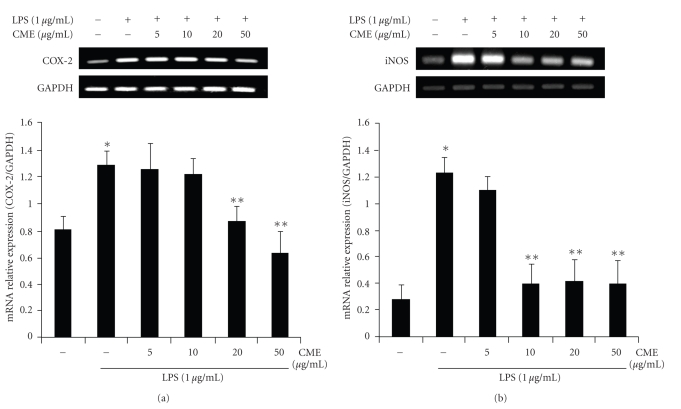
Suppressive effects of CME on the LPS-induced mRNA expression of COX-2 and iNOS in RAW 264.7 cells. The RAW 264.7 cells were pretreated with CME for 1 hour, and then incubated with LPS for 6 hours. The total mRNA was isolated, and the mRNA levels of (a) COX-2 and (b) iNOS were performed by RT-PCR. * indicates an increase in mRNA expression relative to the control (*P* < .05), ** indicates a decrease in mRNA expression relative to the LPS group (*P* < .05).

The expression of iNOS and COX-2 mRNA was examined by RT-PCR to determine if CME has a suppressive effect on the proinflammatory mediators, such as iNOS and COX-2, in the LPS-stimulated RAW 264.7 cells. As shown in Figure 5, upon LPS treatment for 6 h, the mRNA expression of COX-2 increased considerably in RAW 264.7 cells. However, the mRNA expression of COX-2 induced in cells treated with LPS was decreased significantly by 20 *μ*g/mL and 50 *μ*g/mL CME compared to cells treated with LPS alone. The mRNA expression of iNOS was also found to increase in the stimulated LPS macrophage RAW 264.7 cells. However, the LPS-induced iNOS mRNA expression was decreased by 10 *μ*g/mL, 20 *μ*g/mL, and 50 *μ*g/mL CME (Figure 5). This suggests that CME may inhibit the mRNA expression of iNOS and COX-2 at the transcription level in LPS-stimulated RAW 264.7 cells.

### 3.4. CME Decreases LPS-Induced Protein Expression of iNOS and COX-2 in RAW 264.7 Cells

**Figure 6:**
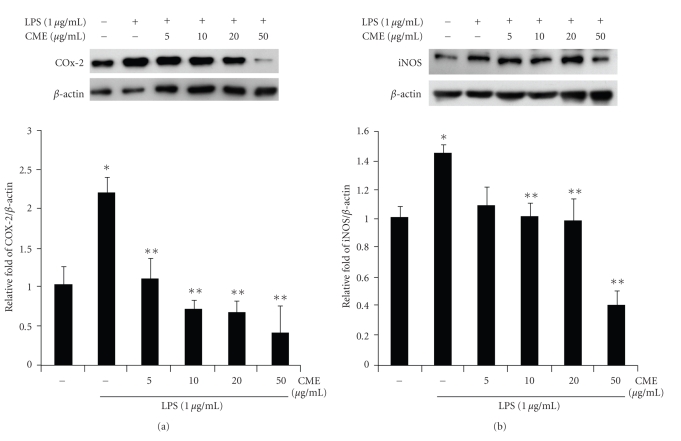
Suppressive effects of CME on the LPS-induced protein expression of COX-2 and iNOS in RAW 264.7 cells. RAW 264.7 cells were pretreated with CME for 1 hour, and then incubated with LPS for 18 hours. The cells were lysed, and the lysates were examined by immunoblotting for the protein of (a) COX-2 and (b) iNOS. * indicates an increase in protein expression relative to the control (*P* < .05), ** indicates a decrease in protein expression relative to the LPS group (*P* < .05).

The effect of CME on the expression of iNOS and COX-2 at the protein levels was examined by western blot analysis.  Figure 6 shows a representative western blot of COX-2 and iNOS in the treated cells. After the LPS treatment for 18 hours, the levels of iNOS and COX-2 protein expression was obviously increased, but a cotreatment with LPS and CME (various concentrations) significantly attenuated the expression of the iNOS and COX-2 proteins in a dose-dependent manner (Figure 6). This suggests that the expression of the iNOS and COX-2 proteins in LPS-induced macrophage RAW 264.7 cells was inhibited by CME.

### 3.5. CME Inhibits I*κ*B Phosphorylation in Cytoplasm

**Figure 7:**
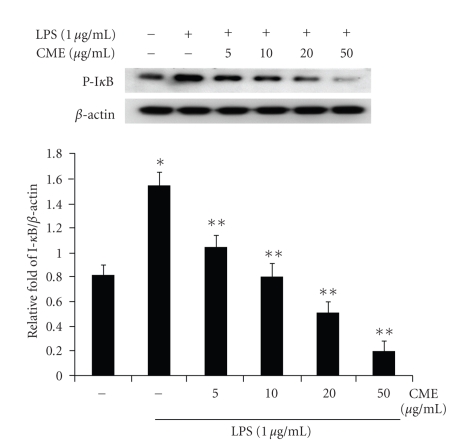
CME suppressed the LPS-induced phosphorylation of I-*κ*B in RAW 264.7 cells. The cells were pretreated with CME at various concentrations for 1 hour and then with LPS for 30 minutes. The cell lysates were analyzed by immunoblotting for the detection of the phosphorylated form of I-*κ*B. * indicates an increase in protein relative to the control (*P* < .05), ** indicates a decrease in protein relative to the LPS group (*P* < .05).

In the NF-*κ*B signal pathway, the phosphorylation and degradation of I*κ*B is a crucial step for NF-*κ*B activation in activated macrophages. This study investigated whether CME inhibits the LPS-induced phosphorylation of I*κ*B and degradation of I*κ*B in RAW 264.7 macrophages by Western blot analysis. As shown in Figure 7, the phosphorylated forms of I*κ*B were barely detectable in the unstimulated RAW 264.7 cells. However, level of I*κ*B phosphorylation was increased significantly after the LPS (1 *μ*g/mL) treatment for 30 minutes. A pretreatment with CME moderately inhibited the LPS-mediated I*κ*B phosphorylation at all CME concentrations. However, CME had no effect on the level of I*κ*B degradation (data not shown).

### 3.6. CME Inhibits NF-*κ*B Activation and Its Nuclear Translocation

**Figure 8:**
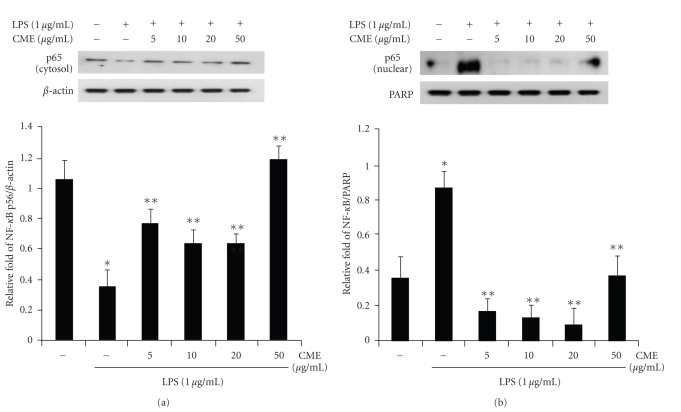
CME suppressed the LPS-induced activation and translocation of NF-*κ*B in RAW 264.7 cells. The cells were pretreated with CME at concentration of 5, 10, 20, and 50 *μ*g/mL for 1 hour and treated with LPS (1 *μ*g/mL). After 30 minutes incubation, the (a) cytosol and (b) nuclear protein fractions were prepared by cell lysis. The fraction proteins were analyzed by immunoblotting for the detection of p65. * indicates an increase in protein relative to the control (*P* < .05), ** indicates a decrease in protein relative to the LPS group (*P* < .05).

p65 of the NF-*κ*B subunit is a major component of NF-*κ*B activated by LPS in macrophages, and the activation of NF-*κ*B is initiated by I*κ*B degradation after I*κ*B-*α* phosphorylation. In this study, Western blot analysis was used to determine if CME inhibited the activation of NF-*κ*B p65 and nuclear translocation of p65 as a result of the inhibition of I*κ*B-*α* phosphorylation. As shown in Figure 8, p65 was less expressed in the cytosol fraction and strongly expressed in the nuclear fraction after exposure to LPS. However, p65 of the cytosol fraction and nuclear fraction were increased and decreased by CME, respectively. Hence, it is hypothesized that CME may block the LPS-induced translocation of p65 from the cytosol to the nucleus by inhibiting I*κ*B phosphorylation.

## 4. Discussion

Herbal medicinal plants or extracts are used as an alternative therapy. *Citrus aurantium L.* has been used as a traditional medicine to treat the inflammatory response. Many studies have reported the pharmaceutical effects of natural herbal products, such as anti-inflammatory, anticancer, and antioxidative effects, and so forth [26–28]. Recent reports suggest that natural herbs have an anti-inflammatory effect by regulating the activation of transcription factors including NF-*κ*B or activator protein-1 (AP-1). In particular, flavonoids, such as epigallocatechin gallate (EGCG), curcumin, and quercetin, have been reported to be potential inhibitors via the NF-*κ*B pathway [29, 30]. Flavonoids are one of the most critical phytochemical compounds in many plants, particularly in the genus Citrus [31]. Citrus-derived flavonoids such as naringenin, nobiletin, and hesperidin have considerable biological activity, including anti-inflammatory effects [32]. Naringenin inhibited several cytokines induced by LPS-stimulated macrophages [33]. Nobiletin suppressed the production of PGE_2_ by downregulating the COX-2 gene in human synovial fibroblasts as well as decreasing the expression of IL-1*α*, IL-1*β*, TNF-*α*, and IL-6 mRNAs in mouse macrophages [34]. Zielińska-Przyjemska and Ignatowicz reported that citrus fruit flavonoids have protective effects against oxidative stress related to inflammation [35].

The present study examined the suppressive effect of CME on the proinflammatory mediators in macrophage RAW 264.7 cells. Therefore, an MTT assay was carried out to examine the cytotoxicity of CME. CME did not exhibit cytotoxicity at any of the concentrations examined. The morphological change in macrophage RAW 264.7 cells induced by the CME treatment in the presence or absence of LPS was also examined. The cells were round without the LPS treatment but became irregularly shaped showing increased spreading and pseudopodia formation after LPS stimulation. The cotreatment with 50 *μ*g/mL CME decreased the degree of cell spreading and pseudopodia formation. Interestingly, this amount of CME also decreased the levels of iNOS and COX-2. 

Inducible enzymes, such as iNOS and COX-2, are critical factors in the inflammatory response, cell proliferation, and skin tumor promotion [21, 36]. In the inflammatory response, iNOS and COX-2 expression are involved in the production of NO and prostaglandin, respectively. The overexpression of iNOS and COX-2 is related to the pathogenesis of a range of disease processes [11, 37]. Previous studies reported that Zedoarondiol isolated from the rhizome of *Curcuma heyneana* inhibits the LPS-induced expression of iNOS and COX-2 with the concomitant reduction of PGE2 and NO production in RAW 264.7 cells [38]. Y.-L. Lin and J.-K. Lin showed that (-)-epigallocatechin-3-gallate (EGCG), one of the flavonoids in green tea, decreased the protein expression of iNOS by reducing the mRNA levels of iNOS through the inhibition of NF-*κ*B binding [39]. Chrysin, a flavonoid contained in propolis and honey, inhibited LPS-induced expression of COX-2 in macrophages by blocking the DNA-binding activity of C/EBP, which plays a major role in inducing the expression of COX-2 [40]. Moreover, the methanol extract of *Citrus reticulate* decreased the production of NO by suppressing the expression of iNOS at the mRNA and protein levels [3]. Therefore, the regulation of iNOS and COX-2 is important in the inflammation response. This study examined the effect of CME on the expression of iNOS and COX-2 at the protein and mRNA levels. The results showed that CME dose dependently suppressed the expression of COX-2 at both the protein and mRNA levels. In addition, the expression of iNOS protein and mRNA in LPS-stimulated macrophage RAW 264.7 cells was suppressed by various concentrations of CME (10, 20, and 50 *μ*g/mL). This shows that CME inhibited the expression of iNOS and COX-2 in LPS-stimulated RAW 264.7 cells at both the protein and mRNA levels. 

Proinflammatory cytokines, such as IL-1*β*, IL-2, IL-6, IL-10, and TNF-*α*, are important mediators in a range of acute and chronic responses to inflammatory diseases [29, 41, 42]. The production of TNF-*α* is essential for the synergistic release of NO in IFN-*γ* and/or LPS-stimulated macrophages. In addition, TNF-*α* is involved in many physiological effects, such as septic shock, inflammation, cachexia, and cytotoxicity. IL-6 is believed to be an endogenous pyrogen that causes fever by triggering metabolic changes in the hypothalamic thermoregulatory center for the duration of the inflammation response [11, 25]. This study investigated the suppressive effect of CME on LPS-induced gene expression of IL-6 and TNF-*α* in macrophage RAW 264.7 cells. The results showed that the mRNA expression of TNF-*α* and IL-6 in LPS-stimulated RAW 264.7 cells was decreased by various concentrations of CME (10, 20, and 50 *μ*g/mL). These results suggest that CME successfully suppressed the expression of TNF-*α* and IL-6 in LPS-stimulated macrophages. 

NF-*κ*B is a major factor regulating the expression of inflammation-induced enzymes and cytokines, such as iNOS, COX-2, TNF-*α*, and IL-6, which include the NF-*κ*B binding sites in their promoters, and has attracted attention as a new target for treating inflammatory diseases [43–46]. Therefore, the suitable regulation of NF-*κ*B may be beneficial in treating many inflammatory disorders. Recently, Jung et al. reported that the root of Panax notoginseng (PN) inhibited the LPS-induced inflammatory mediators, including iNOS and COX-2 by blocking I*κ*B degradation in the cytosol and the nuclear translocation of the NF-*κ*B p65 subunit [12]. Choi et al. showed that nobiletin isolated from the fruit peel of *Citrus sunki *inhibits the expression of the genes involved in inflammation by blocking the DNA-binding activity of NF-*κ*B. However, nobiletin did not affect the LPS-induced phosphorylation and degradation of the I*κ*B-*α* protein or the nuclear translocation of NF-*κ*B [47]. The present study examined whether CME inhibits the LPS-induced degradation and phosphorylation of I*κ*B or the activation and translocation of NF-*κ*B p65. These results showed that CME inhibits significantly the LPS-induced activation and nuclear translocation of NF-*κ*B p65 at various concentrations (5, 10, 20, and 50 *μ*g/mL). Moreover, in RAW 264.7 cells pretreated with CME, the phosphorylation of I*κ*B was inhibited in a dose-dependent manner. However, there were no significant differences in the level of I*κ*B degradation (date not shown). These findings suggest that CME suppresses NF-*κ*B activation and nuclear translocation in LPS-stimulated RAW 264.7 cells by blocking the phosphorylation of I*κ*B. 

In summary, the anti-inflammatory activity of CME was characterized by the suppression of iNOS, COX-2, and various cytokines, such as TNF-*α* and IL-6, through the NF-*κ*B signal pathway. The results showed that CME inhibits the LPS-induced expression of iNOS and COX-2 at the mRNA and protein levels as well as the expression of TNF-*α*, IL-6 transcripts in macrophage RAW 264.7 cells. These suppressive effects are mediated by inhibiting the activation of NF-*κ*B and phosphorylation of I*κ*B. The major transcriptional factors of the target gene, NF-*κ*B, are inactivated by CME. This is important because NF-*κ*B plays a key role in modulating many genes related to the inflammatory response. These results suggest that crude methanol extract of *Citrus aurantium L.* has anti-inflammatory properties, and may be effective as a medicine for preventing inflammation. 

## Figures and Tables

**Table 1:** Primer design for RT-PCR.

Gene	Direction	Sequence (5′-3′)
TNF-*α*	Sense	AGCACAGAAAGCATGATCCG
	Antisense	GTTTGCTACGACGTGGGCTA
IL-6	Sense	CGATGATGCACTTGCAGAAA
	Antisense	TGGAAATTGGGGTAGGAAGG
COX-2	Sense	CCCAGAGCTCCTTTTCAACC
	Antisense	ATTTGGCACATTTCTTCCCC
iNOS	Sense	CTCCCCTCTCTCCCTTTCCT
	Antisense	TGGAAATTGGGGTAGGAAGG
GAPDH	Sense	AAGGGTCATCATCTCTGCCC
	Antisense	GTGATGGCATGGACTGTGGT
